# Quadratic regression analysis for gene discovery and pattern recognition for non-cyclic short time-course microarray experiments

**DOI:** 10.1186/1471-2105-6-106

**Published:** 2005-04-25

**Authors:** Hua Liu, Sergey Tarima, Aaron S Borders, Thomas V Getchell, Marilyn L Getchell, Arnold J Stromberg

**Affiliations:** 1 Department of Statistics, University of Kentucky, Lexington, KY 40506, USA; 2 Department of Physiology, University of Kentucky, Lexington, KY 40536, USA; 3 Department of Anatomy and Neurobiology, University of Kentucky, Lexington, KY 40536, USA; 4 Sanders-Brown Center on Aging, University of Kentucky, Lexington, KY 40536, USA

## Abstract

**Background:**

Cluster analyses are used to analyze microarray time-course data for gene discovery and pattern recognition. However, in general, these methods do not take advantage of the fact that time is a continuous variable, and existing clustering methods often group biologically unrelated genes together.

**Results:**

We propose a quadratic regression method for identification of differentially expressed genes and classification of genes based on their temporal expression profiles for non-cyclic short time-course microarray data. This method treats time as a continuous variable, therefore preserves actual time information. We applied this method to a microarray time-course study of gene expression at short time intervals following deafferentation of olfactory receptor neurons. Nine regression patterns have been identified and shown to fit gene expression profiles better than k-means clusters. EASE analysis identified over-represented functional groups in each regression pattern and each k-means cluster, which further demonstrated that the regression method provided more biologically meaningful classifications of gene expression profiles than the k-means clustering method. Comparison with Peddada et al.'s order-restricted inference method showed that our method provides a different perspective on the temporal gene profiles. Reliability study indicates that regression patterns have the highest reliabilities.

**Conclusion:**

Our results demonstrate that the proposed quadratic regression method improves gene discovery and pattern recognition for non-cyclic short time-course microarray data. With a freely accessible Excel macro, investigators can readily apply this method to their microarray data.

## Background

Microarray time-course experiments allow researchers to explore the temporal expression profiles for thousands of genes simultaneously. The premise for pattern analysis is that genes sharing similar expression profiles might be functionally related or co-regulated [1]. Due to the large number of genes involved and the complexity of gene regulatory networks, clustering analyses are popular for analyzing microarray time-course data. Heuristic-based cluster analyses group genes based on distance measures; the most commonly used methods include hierarchical clustering [2], k-means clustering [3], self-organizing maps [4], and support vector machines [5]. Due to the lack of statistical properties of these heuristic-based clustering methods, statistical models, especially analysis of variance (ANOVA) models and mixed models are often implemented as a precursor to clustering to ensure the genes used for clustering are statistically meaningful [6,7]. Only genes identified to be significantly regulated by statistical models are used for further clustering. Fitting statistical models prior to clustering usually dramatically reduces the number of genes used for clustering, which in general will improve the performance of the clustering method. An alternative way of clustering is statistical model-based clustering methods, which assume that the data is from a mixture of probability distributions such as multivariate normal distributions and describe each cluster using a probabilistic model [8,9].

In microarray time-course studies, time dependency of gene expression levels is usually of primary interest. Since time can affect the gene expression levels, it is important to preserve time information in time-course data analysis. However, most methods for analyzing microarray time-course data treat time as a nominal variable rather than a continuous variable, and thus ignore the actual times at which these points were sampled. Peddada et al. (2003) proposed a method for gene selection and clustering using order-restricted inference, which preserves the ordering of time but treats time as nominal [1]. Recently, a number of algorithms treating time as a continuous variable have been introduced. Xu et al. (2002) applied a piecewise regression model to identify differentially expressed genes [10]. Both Luan and Li (2003) and Bar-Joseph et al (2003) proposed B-splines based approaches [11,12], which are appropriate for microarray data with relatively long time-course, but their application to short time-course data is questionable. New methods for analyzing short time-course microarray data are needed [13].

In this paper, we propose a model-based approach, step down quadratic regression, for gene identification and pattern recognition in non-cyclic short time-course microarray data. This approach takes into account time information because time is treated as a continuous variable. It is performed by initially fitting a quadratic regression model to each gene; a linear regression model will be fit to the gene if the quadratic term is determined to have no statistically significant relationship with time. Significance of gene differential expression and classification of gene expression patterns can be determined based on relevant F-statistics and least squares estimates. Major advantages of our approach are that it not only preserves the ordering of time but also utilizes the actual times at which they were sampled; it identifies differentially expressed genes and classifies these genes based on their temporal expression profiles; and the temporal expression patterns discovered are readily understandable and biologically meaningful. A free Excel macro for applying this method is available at [14]. The proposed quadratic regression method is applied to a microarray time-course study of olfactory receptor neurons [15]. Biologically meaningful temporal expression patterns have been obtained and shown to be more effective classifications than ANOVA-protected k-means clusters. Comparison with Peddada et al.'s order-restricted inference method [1] showed that our method provides a different and interesting insight into the temporal gene profiles. Reliabilities of the results from all 3 methods were assessed using a bootstrap method [16] and regression patterns were shown to have the highest reliabilities.

## Results

### Step-down quadratic regression

We propose a step-down quadratic regression method for gene discovery and pattern recognition for non-cyclic short time-course microarray experiment. The first step is to fit the following quadratic regression model to the j^th ^gene:

*y*_*ij *_= *β*_0*j *_+ *β*_1*j*_*x *+ *β*_2*j*_*x*^2 ^+ *ε*_*ij *_    (1)

where *y*_*ij *_denotes the expression of the j^th ^gene at the i^th ^replication, *x *denotes time, *β*_0*j *_is the mean expression of the j^th ^gene at *x *= 0, *β*_1*j *_is the linear effect parameter of the j^th ^gene, *β*_2*j *_is the quadratic effect parameter of the j^th ^gene, and, *ε*_*ij *_is the random error associated with the expression of the j^th ^gene at the i^th ^replication and is assumed to be independently distributed normal with mean 0 and variance . Two levels of significance, *α*_0 _and *α*_1_, need to be pre-specified, where *α*_0 _to is recommended to be small to reduce the false positive rate in the gene discovery and *α*_1 _less stringent to control pattern classification. *α*_0 _could be chosen using various multiple testing p-value adjustment procedures, for example, False Discovery Rate (FDR) [17]. The temporal gene expression patterns can be determined as follows:

1. If overall model (1) p-value >*α*_0_, the j^th ^gene is considered to have no significant differential expression over time. The expression pattern of the gene is "flat".

2. If overall model (1) p-value ≤ *α*_0_, the j^th ^gene will be considered to have significant differential expression over time. The patterns are then determined based on the p-values obtained from F tests (Table 1).

a. If both p-value of quadratic effect ≤ *α*_1 _and p-value of linear effect ≤ *α*_1_, the j^th ^gene is considered to be significant in both the quadratic and linear terms. The expression pattern of the gene is "quadratic-linear".

b. If p-value of quadratic effect ≤ *α*_1 _and p-value of linear effect >*α*_1_, the j^th ^gene is considered to be significant only in the quadratic term. The expression pattern of the gene is "quadratic".

c. If p-value of quadratic effect *>α*_1_, the j^th ^gene is considered to be non-significant in the quadratic term. The quadratic term will be dropped and a linear regression model will be fitted to the gene:

*y*_*ij *_= *β*_0*j *_+ *β*_1*j*_*x *+ *ε*_*ij *_    (2)

From fitting model (2),

• If p-value of linear effect ≤ *α*_1_, the j^th ^gene is considered to be significant in the linear term. The expression pattern of the gene is "linear".

• If p-value of linear effect *>α*_1_, the j^th ^gene is considered to be non-significant in the linear term. The expression pattern of the gene is "flat".

**Figure 1:**
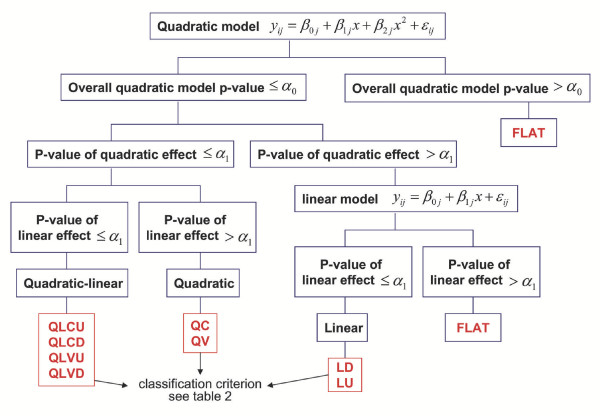
**Flow chart of the quadratic regression method**. The gene selection and pattern classification procedure of our quadratic regression method. *y*_*ij *_is the expression level; *x *is time; *β*_0*j*_, *β*_1*j*_, and *β*_2*j *_are the parameters of intercept, linear effect, and quadratic effect, respectively; *ε*_*ij *_is the random error. Among the 9 regression patterns, FLAT stands for no statistically significant differential expression over time; LU stands for linear up; LD stands for linear down; QC stands for quadratic concave; QV stands for quadratic convex; QLCU stands for quadratic linear concave up; QLCD stands for quadratic linear concave down; QLVU stands for quadratic linear convex up; QLVD stands for quadratic linear convex down.

The four expression patterns described above can be furthered classified into 9 patterns according to the up/down regulation of the gene expression based on the least-squares estimates  and  and the predicted signals (Table 2). A flow chart for the above procedure is shown in Figure 1. This procedure can be easily applied using the Excel macro available at [14].

### Application of the quadratic regression method

**Figure 2:**
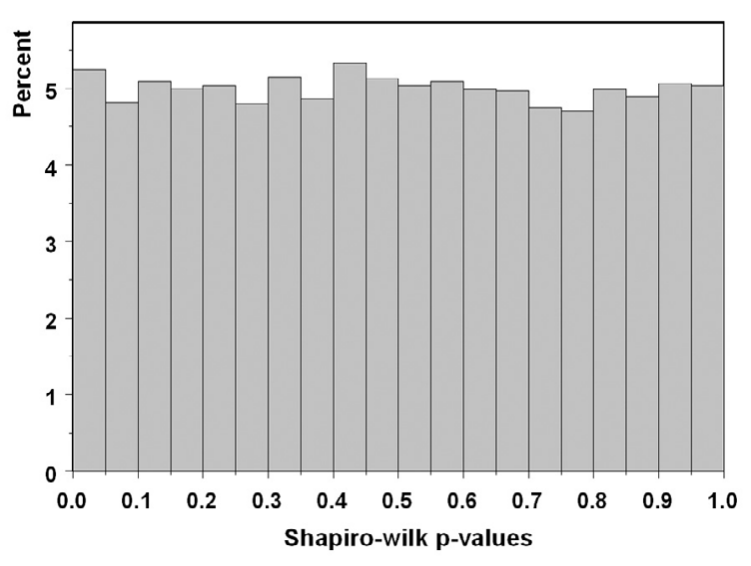
**Histogram of the Shapiro-Wilk p-values for normality test**. The Shapiro-Wilk statistic was applied to the olfactory receptor neuron data for normality test. The horizontal axis is the Shapiro-Wilk p-values, and the vertical axis is the corresponding percentages. This histogram indicates that most of the 3834 present genes do not have a significant departure from the normality.

**Figure 3:**
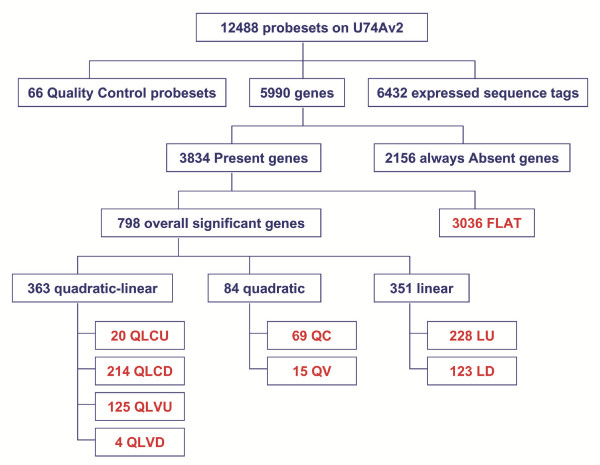
**Flow chart of the filtering steps and quadratic regression analysis on the olfactory receptor neuron data**. Our regression method is applied to the olfactory receptor neuron data. At first, Affymetrix quality controls, expressed sequence tags, and genes which have "A" calls across all chips were removed from the analysis. Nine regression patterns were identified among 3834 remaining genes (colored in red). FLAT stands for no statistically significant differential expression over time detected by the regression method; LU stands for linear up regulated regression pattern; LD stands for linear down regulated regression pattern; QC stands for quadratic concave regulated regression pattern; QV stands for quadratic convex regulated regression pattern; QLCU stands for quadratic linear concave up regulated regression pattern; QLCD stands for quadratic linear concave down regulated regression pattern; QLVU stands for quadratic linear convex up regulated regression pattern; QLVD stands for quadratic linear convex down regulated regression pattern.

**Figure 4:**
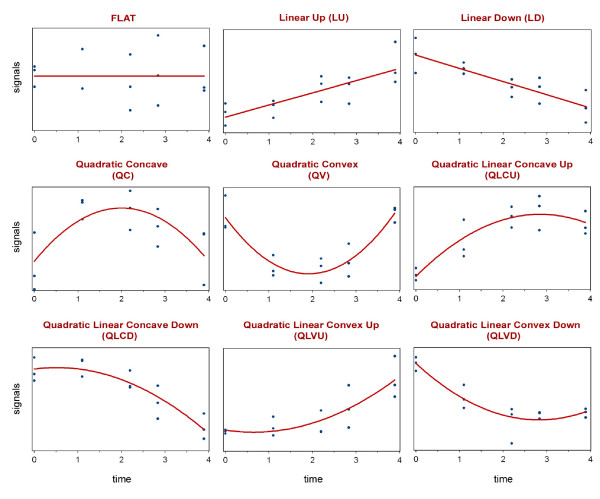
**An illustration of the nine temporal expression patterns identified by the quadratic regression method**. The horizontal axis is the log transformation of time. The vertical axis is the hybridization signals obtained from the microarrays. The blue dots are the hybridization signals. The red line or curve is the fitted regression pattern. FLAT stands for no statistically significant differential expression over time detected by the regression method; LU stands for linear up regulated regression pattern; LD stands for linear down regulated regression pattern; QC stands for quadratic concave regulated regression pattern; QV stands for quadratic convex regulated regression pattern; QLCU stands for quadratic linear concave up regulated regression pattern; QLCD stands for quadratic linear concave down regulated regression pattern; QLVU stands for quadratic linear convex up regulated regression pattern; QLVD stands for quadratic linear convex down regulated regression pattern. The corresponding gene symbols are: FLAT. *Cldn11*; LU. *Gba*; LD. *Col6a3*; QC. *Rab18*; QV. *unknown*; QLCU. *Psmb6*; QLCD. *Hnrpa2b1*; QLVU. *Tyrobp*; QLVD. *Acvr2b*.

Normality test based on Shapiro-Wilk statistics [18] suggested that most of the 3834 present genes in the olfactory receptor neuron data do not have a significant departure from the normal distribution (Figure 2). Therefore the quadratic regression method with normality assumption was applied to the data of 3834 present genes (Figure 3), where *α*_0 _was chosen to be 0.01 and *α*_1 _to be 0.05. 798 genes were determined to have significant differential expression over time at level 0.01. Examples of 9 regression patterns are shown in Figure 4.

### Comparison with Peddada et al.'s method

**Figure 5:**
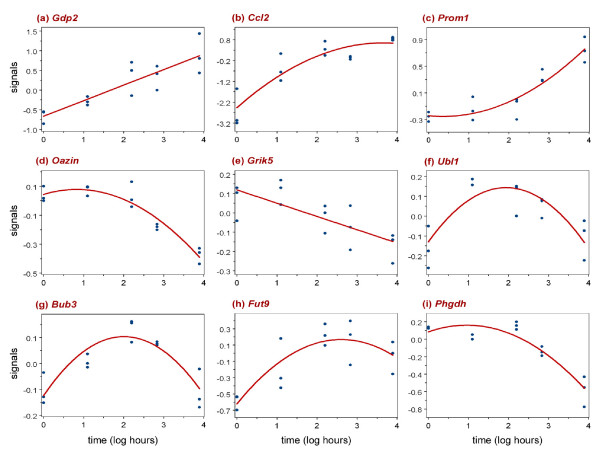
**Examples of genes in the comparison among regression patterns and Peddada et al.'s profiles**. a. *Gdp2*; b. *Ccl2*; c. *Prom1*; d. *Oazin*; e. *Grik5*; f. *Ubl1*; g. *Bub3*; h. *Fut9*; i. *Phgdh*. The genes in a, b, and c all have Peddada et al.'s MI profile, but are in 3 different regression patterns LU, QLCU and QLVU, the difference among the temporal profiles of these genes is the rate of increase. The genes in d, e, and f all have Peddada et al.'s UD2 profile, but are in 3 different regression patterns QLCD, LD, and QC. The genes in g, h, and i all have Peddada et al.'s UD3 profile, but are in 3 different regression patterns QC, QLCU, and QLCD. The differences among d, e, f and among g, h, i are due to the relationship among all time points and with the maximum. The horizontal axis is the log transformation of time. The blue dots are the signals. The red line or curve is the fitted regression pattern.

Peddada et al.'s method [1] was applied to the expression data of 3834 present genes with 8 pre-specified profiles: monotone increasing (MI); monotone decreasing (MD); 3 up-down profiles with maximum at the second, third, forth time point (UD2, UD3, UD4); and 3 down-up profiles with maximum at the second, third, forth time point (DU2, DU3, DU4). Based on 4000 bootstrap, 379 genes were classified into one of the 8 pre-specified profiles at significance level 0.01. This indicates that Peddada et al.'s method might be relatively more conservative than regression method by selecting much fewer genes at significance level 0.01. Comparisons of Peddada et al.'s profiles and regression patterns are listed in Table 3. We observe that the majority of genes in MI are in LU, similarly for MD and LD, UD2 and QLCD, and DU2 and QLVU. However, each of the Peddada et al.'s profiles contains a mixture of regression patterns, and vice versa. This is reasonable because even though both methods perform gene selection and classification, they are aimed at different aspects of the temporal profiles. For example, Peddada et al.'s MI profile contains regression patterns LU, QLCU and QLVU. Although the gene expression level is increasing monotonically over time, the regression method gives more information on how it is increased: constantly (LU, Figure 5a, *Gdp2*), increases faster then slower (QLCU, Figure 5b, *Ccl2*), or increases slower then faster (QLVU, Figure 5c, *Prom1*). Peddada et al.'s UD2 profile contains genes that are first up-regulated then down-regulated with maximum at the second time points, which could be classified as regression pattern QLCD in general (Figure 5d, *Oazin*), but it could also be classified as LD if the expression levels of all time points are close to a line (Figure 5e, *Grik5*); or classified as QC if the expression profile is close to quadratic (Figure 5f, *Ubl1*); or classified as QLCU if the expression levels of last 4 time points are much closer than those of the first time point. Similarly, Peddada et al.'s UD3 profile could be classified as regression patterns QC, QLCU, and QLCD (Figure 5g, *Bub3*; 5h, *Fut9*; 5i, *Phgdh*).

### Comparison with ANOVA-protected k-means clustering

ANOVA-protected k-means clustering was applied to the expression signals of 3834 present genes. Out of 3834 present genes, 770 were identified to be differentially expressed over time by one way ANOVA (overall model p-value ≤ 0.01). These 770 genes were used for classification by k-means clustering with k = 9 and the distance measure being Pearson correlation coefficient (Table 4).

**Figure 6:**
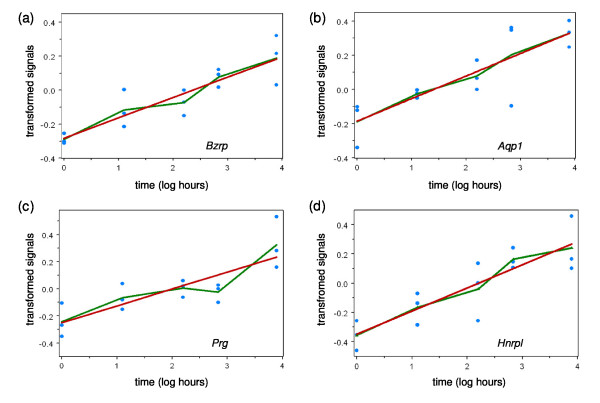
**Examples of genes with the same LU regression pattern but in different k-means clusters**. a. *Bzrp *is an example from k-means cluster K2; b. *Aqp1 *is an example from k-means cluster K5; c. *Prg *is an example from k-means cluster K6; d. *Hnrpl *is an example from k-means cluster K8. These 4 genes are all identified to have the LU regression pattern, but in 4 different k-means clusters. The LU regression pattern is clearly a good fit to the temporal expression profiles of these 4 genes. The horizontal axis is the log transformation of time. The blue dots are the signals. The green line is the connection of the mean signal at each time point. The red line is the LU regression pattern.

**Figure 7:**
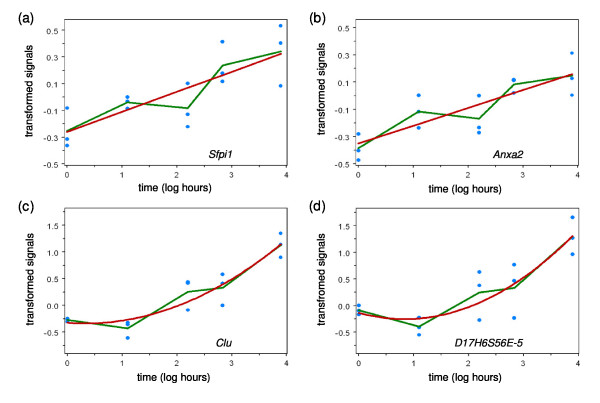
**Examples of genes with similar expression patterns in terms of mean signal and regression**. a. *Sfpi1 *is an example from k-means cluster K2; b. *Anxa2 *is an example from k-means cluster K8; c. *Clu *is an example from k-means cluster K5; d. *D17H6S56E-5 *is an example from k-means cluster K6. a and b are examples of genes with the same up-down-up-up pattern (up-regulated at the second time point, down-regulated at the third time point, then up-regulated at the last two time points) in terms of mean transformed signals (green lines). They also have the same LU regression pattern, but are in different k-means clusters. c and d are examples of genes with the same down-up-down-up pattern in terms of mean transformed signals (green lines). They also have the same QLVU regression pattern, but are in different k-means clusters. Clearly, the regression method provides better classification of the temporal expression profiles of these genes than the k-means clustering method. The horizontal axis is the log transformation of time. The blue dots are the signals. The green line is the connection of the mean signal at each time point. The red line or curve is the fitted regression pattern.

**Figure 8:**
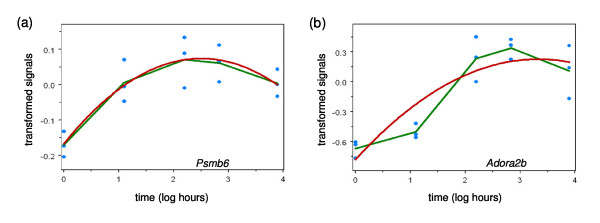
**Examples of genes with the same regression pattern but different onset of differential expression**. a. *Psmb6 *is an example in k-means cluster K8; b. *Adora2b *is an example in k-means cluster K5. *Adora2b *clearly starts differential expression later than *Psmb6*. After the onset point (first time point for *Psmb6 *and second time point for *Adora2b*), these two genes show similar upward regulation. The regression method classifies these two genes into the same group (QLCU regression pattern), but k-means clustering method does not. The horizontal axis is the log transformation of time. The blue dots are the signals. The green line is the connection of the mean signal at each time point. The red curve is the QLCU regression pattern.

In order to make the regression patterns comparable with the k-means clusters, the quadratic regression method was applied to the 770 ANOVA significant genes. Table 4 shows the number of genes in common when comparing each regression pattern with each k-means cluster. An example of a good match between regression patterns and k-means clusters is the QLCD regression pattern and k-means cluster K1. However, in most cases, k-means clusters contain a mixture of regression patterns and the regression patterns are separated into different k-means clusters. For example, genes that have the LU regression pattern are split into 4 k-means clusters (Figure 6a, *Bzrp*; 6b, *Aqp1*; 6c, *Prg*; 6d, *Hnrpl*). The similarity of the temporal expression profiles in Figure 6 indicates that it might be more appropriate to classify these genes into the same group, which occurs using the proposed regression method. Examples in Figure 7 show that some k-means clusters are also mixtures of expression profiles in terms of the mean signals (green lines). For example, a down-up-down-up pattern (down-regulated at the second time point, up-regulated at the third time point, etc, in terms of mean signals) appeared in both k-means clusters K5 and K6 (green lines), but are identified to have QLVU regression pattern (Figure 7c, *Clu*; and 7d, *D17H6S56E-5*); similarly see Figure 7a and 7b (a, *Sfpi1*; and b, *Anxa2*). Once again, the regression method provides better classification. Figure 8 is an example of genes with similar expression patterns but different initial starting time of the differential expression (Figure 8a, *Psmb6*; 8b, *Adora2b*). *Adora2b *clearly starts differential expression later than *Psmb6 *(see the blue dots in Figure 8). After the initial starting point (first time point for *Psmb6 *and second time point for *Adora2b*), these two genes show similar upward regulation. These two genes were classified into the same regression group, but in different k-means clusters. Based on the above analysis, our regression method is demonstrated to be more appropriate for the classification of temporal gene expression profiles than k-means method.

### EASE functional analysis on regression patterns and k-means clusters

To further explore the effectiveness of the regression method on gene classification, EASE (Expression Analysis Systematic Explorer) software was used to examine the potential relationship between the biological functions of the genes and their expression patterns [19]. EASE calculates EASE scores (Jackknife one-sided Fisher exact p-values) to identify over-represented gene categories within lists of genes. EASE analysis was applied to each of the 9 regression patterns and 9 k-means clusters that were obtained from the classification of 770 ANOVA significant genes (Table 4). The results are summarized (see Additional file 1), with part of the information shown in Tables 5 and 6. The EASE analysis demonstrates that the proposed regression method is more effective for gene classification than the k-means clustering method. Almost all of the regression patterns contain genes mainly from one biological process. For example, LU has 9 over-represented gene categories, 8 of which are involved in immune regulation (Table 5). The majority of the LU and QLVU gene categories are in the immune regulation category. This suggests that there exist multiple regulatory mechanisms within the immune regulation. The immune regulation in QLVU appears to be a more complex regulatory mechanism for the initial up-regulation of these genes due to the slow upward regulation at early time points of this regression pattern (Figure 5c). The EASE results for the k-means clusters shows that the over-represented gene categories of most k-means clusters are involved in more than one biological process, for example, k-means cluster K5 contains 9 over-represented gene categories, 3 involved in immune regulation, 2 involved in cell death, etc. Notice that the immune regulation category is represented in 4 k-means clusters, which suggests that the immune regulation category is more consolidated in regression patterns than in k-means clusters (Table 6). Also, by comparing EASE scores in Tables 5 and 6, one can see that the over-represented gene categories in the regression patterns have, in general, smaller EASE scores than those in the k-means clusters, which further indicates the greater effectiveness of the regression method in pattern classification.

### Reliability analysis

**Figure 9:**
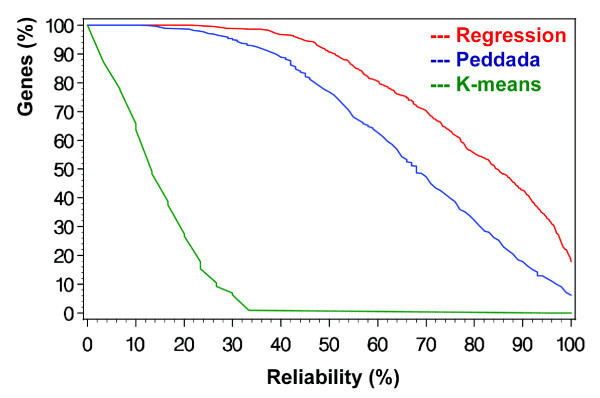
**Reliability curves of regression patterns, Peddada et al.'s profiles, and k-means clusters**. The horizontal axis is the reliability (percentage of agreement of the bootstrap results with the original result), and the vertical axis is the corresponding percentage of genes. The regression patterns show the highest reliability, and k-means clusters show the lowest reliability.

Kerr and Churchill (2001) introduced a bootstrap technique to assess the stability of clustering results [16]. We applied the same idea here to assess the reliability of regression patterns, Peddada et al.'s profiles, and k-means clusters. All 3 pattern classification methods were performed on the expression data of 770 ANOVA significant genes to make the results comparable. The reliability curves show that regression patterns have the highest reliability, and k-means clusters have the lowest reliability (Figure 9). This suggests that the regression method provides relatively more stable pattern classifications.

### Simulation study

We investigated the false positive rate (gene specific) of our method via a simulation study. The data were generated randomly from N(0,1), containing expression signals of 10000 "null" genes (no gene differentially expressed over time), with 5 time points and 3 replications per time point per gene. 50 of such data were generated. The regression approach was applied to each gene in each simulated data at *α*_0 _= 0.01 and the numbers of significant genes in each of the 50 data were obtained. The average proportion of significance (average false positive rate) is 1.01% with standard deviation 0.01%. This demonstrates that the false positive rate of the regression method is accurate because 1% of 10000 genes would be expected to be significant at 0.01 level by chance. The false positive rates of the regression patterns LU, LD, QC, QV are all approximately equal to 1/6 of the average false positives, and those of QLCU, QLCD, QLVU, and QLVD are all approximately equal to 1/12 of the average false positives.

## Discussion

The proposed step-down quadratic regression method is an effective statistical approach for gene discovery and pattern recognition. It utilizes the actual time information, and provides biologically meaningful classification of temporal gene expression profiles. Furthermore, it does not require replication at each time point, which ANOVA-type methods do require. Also, this method can identify genes with subtle changes over time and therefore discover genes that might be undetectable by other methods, eg, ANOVA-type methods. However, there are several limitations to this method. Firstly, it is designed to fit time-course data with a small number of time points. We recommend this method when there are 4 to 10 time points in the data. For an experiment with more time points, spline-type methods [11,12] could be a possible choice; for an experiment with 2 or 3 time points, ANOVA-type method is recommended. Secondly, the 9 regression patterns are rather limited considering the complexity of gene regulatory networks. For example, certain proportion of genes show cubic, "M", and "W" shaped patterns in 211 regression FLAT genes which are ANOVA significant (Table 4). These patterns could be caused by random chance, but they could also be real patterns. Fitting a higher order polynomial regression model may discover these types of genes profiles. Theoretically, one could fit a 4^th^-order polynomial regression model to this data (the highest order of the polynomial one can fit is the number of time points minus one). The model with 4^th^-order polynomial will work similarly to connecting the mean at each time point, therefore will provide a good fit to the data with smallest R^2 ^and minimum Mean Squared Error, compared with lower-order polynomials. However, the purpose of pattern analysis is to cluster the data instead of fitting models, so the quadratic fit is useful even though the goodness of fit may not be great. Also, the use of high-order polynomials (higher than the second-order) should be avoided if possible [20], particularly in cases such as this where the regression coefficients are used primarily for classification. Another issue is the transformation of the experimental time. Transformation should be considered when the sampling time is unequally spaced. The choice for the type of transformation (log-transformation, square-root transformation, etc) is not critical because the resulting pattern classification will in general not be impacted.

In the reliability curves, at 95% reliability, regression patterns, Peddada et al.'s profiles, and k-means clusters have 33%, 12%, and 0% of genes, respectively; and at 80% reliability, the percentage of genes are 55%, 32%, and 0%, respectively (Figure 9). Even though the regression patterns have the highest reliability, only 33% of genes have 95% reliabilities. We examined the overall model (1) p-values of 770 genes by the regression method and found that genes that have the smallest overall model (1) p-values all have 95% reliabilities. This suggests that we could reduce the level of significance *α*_0 _to increase the stability of regression patterns. *α*_0 _could be reduced using various multiple testing p-value adjustment procedures, for example, Westfall and Young's step down method [21], and False Discovery Rate (FDR) [17]. Application of the FDR method can be done as follows (assuming FDR is controlled at level of *α*): let *p*_(1) _<*p*_(2) _< … <*p*_(*m*) _be the ordered overall model (1) p-values, start from the largest p-value *p*_(*m*)_, compare each *p*_(*i*) _with α *i/*m*; let k be the largest *i *that *p*_(*k*) _≤ α *k/*m*, conclude *p*_(1)_, …, *p*_(*k*) _to be significant.

Both our quadratic regression method and Peddada et al.'s method serve the same overall goal: gene selection and classification. Peddada et al.'s method provides more choices of temporal profiles than our method. While our regression method offers less choice of patterns, it may provide deeper insight into the gene expression profiles than Peddada et al.'s method. Our method distinguishes patterns with different rates of change and provides more information on the relative relationship among the expression levels of all time points. For example, specifying a profile of up-down with maximum at one time point does not provide much information on the relative relationships among other time points (Figure 5). A further refinement of Peddada et al.'s method may provide such information about the relationship of other time points besides the maximum/minimum. However, it is less likely to separate the patterns in Figure 5a, b, and 5c by their method. Another fact is that Peddada et al.'s method provides exactly the location of the maximum/minimum, whereas our method provides the neighborhood of the location of the maximum/minimum. Furthermore, their method is based on bootstrap, which is computationally intensive. The result of their method, for example, the reliability curves, might be improved by applying more bootstrap, which is 4000 in this paper due to the computational difficulties and time constraints. Moreover, their method depends on the ordering of time but not the actual time at which the samples were taken, whereas the regression method accounts for both.

K-means is an iterative clustering algorithm [22]. The first step of this method is to randomly assign the data points to the k clusters. Next, the distance to the center of each cluster is calculated for each data point, and the data point is moved into the closest cluster. This step will be repeated until no data point is moving from one cluster to another. In k-means, the number of clusters, k, needs to be pre-specified. Researchers usually choose several different k and find the one which has the most biologically meaningful clusters. There are methods of finding the "optimal" k, for example, Bayesian Information Criterion [23]. In this paper, k was arbitrarily chosen to be 9. Since the k-means clustering does not perform well (Table 4; Figures 6, 7, and 8), we investigated different choice of k based on the Bayesian Information Criterion and identified that the "optimal" k is 15. However, as we examined these 15 k-means clusters, the pattern classification does not seem to be improved, the same problem exists as with k = 9. For example, *Prom1*, *Clu*, and *D17H6S56E-5 *(Figure 5c, Figure 7c and 7d) all have similar temporal profiles and are all classified to be QLVU, but they were separated into 3 of the 15 k-means clusters. This could be related to the distance measure used (Pearson correlation coefficient). As we discovered, genes in the same cluster do not necessarily have higher correlation than genes in different clusters. For example, *Sfpi1 *and *Anxa2 *(Figure 7a and 7b) are highly correlated (Pearson correlation coefficient is 0.9934) and their expression patterns are similar, but they are in different k-means clusters. A possible reason might be that the time-course in olfactory receptor neuron data is too short for correlation to perform well. Even though there are a total of 15 observations for each gene, correlation calculations are based on the 5 mean signals, which could be too few to describe the relationship between temporal profiles. There is also concern about using correlation as the distance measure. A large correlation coefficient does not necessarily indicate two similarly shaped profiles, nor does a small correlation coefficient necessarily indicate differently shaped profiles [1].

A number of regression algorithms have been proposed recently, which treat time as a continuous variable. Several of them are based on cubic B-splines [11,12]. B-splines are defined as a linear combination of a set of basis polynomials. In order to fit cubic B-splines to time-course data, the entire duration of experimental time needs to be divided into several segments by "knots" (the point to separate segments), and each segment will be fit by cubic polynomial. The successful application of these methods to microarray time-course data depends heavily on having a relatively large numbers of time points. The B-spline based methods will not be effective when there are a small number of time points in the time-course experiment [13]. For a data with 5 time points, cubic B-spline type methods would not be appropriate because it is recommended that there should be at least 4 or 5 experimental time points in each segment [24]. Xu et al used a piecewise quadratic regression model to identify differentially expressed genes [10]. In their approach, expression levels at 0 hour and 2 hours after treatment are fit differently from the rest of time points after treatment. Although appropriate for their data, their method cannot be applied to the dataset used in this paper.

The quadratic regression method that we applied to the olfactory receptor neuron data relies on the normality assumption. This is supported by the result of the Shapiro-Wilk normality test, which indicates that most of the genes used for the analysis follow a normal distribution. This might be due to the fact that we removed genes that are called "A" (absent) by Affymetrix across all chips. "A" calls are often assigned to low expression signals, which tend to be non-normal in general. Therefore removing genes with a high proportion of "A" calls may reduce the possibility of violation of the normality assumption, which will then make the test based on distributional assumption more likely to be valid, and thus avoid computational intensive resampling procedures, for example, bootstrap and permutation. If desired, experimenters could also try various types of data transformation to make their data closer to normal when the data are shown to have large departure from normality. However, the log transformation performed on the olfactory receptor neuron data was not to reduce the possible non-normality, but solely to make a fair comparison of our regression method and k-means method because it is the default transformation in Genespring. When the normality assumption (*ε*_*ij *_~ *N*(0,)) does not hold, the bootstrap method [25] can be used to avoid the distributional assumption. For an experiment with m genes, T time points, and r replications per time point, the bootstrap procedure can be performed in the following way: form the data into a matrix of m × rT, each column in the matrix contains expressions of m genes in one chip and each row contains rT expressions of one gene; randomly draw rT columns with replacement to form a bootstrap sample; apply step-down quadratic regression procedure to the bootstrap sample to obtain F statistics from F tests; repeat the above steps 1000 times to form a bootstrap F distribution for each gene; claim a gene to be significance at level of *α *if its observed F statistics is greater than the upper (*α */ 2)^*th*^percentile or less than the lower (*α */ 2)^*th *^percentile of its bootstrap F distribution. One concern about using bootstrap here is that the bootstrap F distribution might be too discrete due to the small number of time points. However, the fact that we are bootstrapping both the explanatory and response variables mitigates this issue by using all the data points, not just the time points. Additionally, in a small simulation study, we observed that the bootstrap F distribution is rather smooth (result not shown).

## Conclusion

The proposed step-down quadratic regression approach is shown to be effective for gene discovery and pattern recognition for non-cyclic short time-course microarray experiment. Major advantages of this method are that it preserves the actual time information, and provides a useful tool for gene identification and pattern recognition. The nine regression patterns, obtained when applied to the olfactory receptor neuron data, are shown to be more reasonable classifications compared to ANOVA-protected k-means clustering method. EASE analysis further showed that our regression patterns are more biologically meaningful than the k-means clusters. Comparison with Peddada et al.'s method showed that our method provides a different perspective on the temporal gene profiles. Reliability study indicates that regression patterns are most reliable. In conclusion, this method should improve gene discovery and pattern recognition for microarray time-course data. With the freely accessible Excel macro, investigators can readily apply this method to their research data.

## Methods

### ANOVA-protected k-means clustering

One-way ANOVA model *y*_*ijk *_= *μ*_*j *_+ *τ*_*k *_+ *ε*_*ijk *_was fitted to each gene in SAS v9, where *y*_*ijk *_denotes the gene expression level of the j^th ^gene at the i^th ^replication of the k^th ^time point, *μ*_*j *_denotes the overall mean signal of the j^th ^gene, *τ*_*k *_denotes the effect of k^th ^time point, *ε*_*ijk *_denotes the random error associated with the i^th ^replication at the k^th ^time point of the j^th ^gene and is assumed to be independently distributed normal with mean 0 and variance . Genes that have overall ANOVA model p-values ≤ *α*_0 _will be used for k-means clustering. K-means clustering was performed in Genespring V6.1 (Silicon Genetics. Redwood City, CA) with k = 9. The similarity measure was chosen to be Pearson correlation coefficient, which was calculated from vectors of length 5 containing mean signals of 3 replications at each of the 5 time points. 500 additional random clusters were tested and the best clusters were selected by the software.

### EASE functional analysis

EASE software was used to identify the over-represented categories of genes [19]. Gene Ontology Biological Process was chosen as the categorization system in EASE analysis. A functional gene category with an EASE score of less than 0.05 is considered to be over-represented. The EASE software is available at: .

### Data description

The data used here are from a study of olfactory receptor neurons [15]. The goal is to investigate the induction of gene regulation at short time intervals following deafferentation of olfactory receptor neurons by target ablation at 2, 8, 16, and 48 hrs compared with the sham control. Total RNA was isolated from 3 male littermate mice per time point. Following hybridization with Affymetrix GeneChips MGU74Av2, 3 chips per time point, the signals were generated by GeneChip Analysis Suite v5.0. The data was filtered before statistical tests were performed. First, 66 Affymetrix quality control probesets and 6432 expressed sequence tags were removed. Next, the absent call (A) provided by Affymetrix was considered. 2156 genes that are called "A" across all 15 chips were removed from the data. The remaining 3834 present genes were used for the regression analysis (Figure 3). The hybridization signals of these 3834 genes were log-transformed in Genespring. Because the time points in this experiment are not equally spaced, ln(t+1) transformation was performed to each of the 5 time points, where t stands for the time point.

## List of abbreviations

ANOVA: analysis of variance.

LD: linear down regulated regression pattern.

LU: linear up regulated regression pattern.

QC: quadratic concave regulated regression pattern.

QV: quadratic convex regulated regression pattern.

QLCD: quadratic-linear concave down regulated regression pattern.

QLCU: quadratic-linear concave up regulated regression pattern.

QLVD: quadratic-linear convex down regulated regression pattern.

QLVU: quadratic-linear convex up regulated regression pattern.

MI: monotone increasing.

MD: monotone decreasing.

UD2/UD3/UD4: up-down with maximum at the second/third/forth time point.

DU2/DU3/DU4: down-up with maximum at the second/third/forth time point.

## Authors' contributions

HL conducted the statistical analyses and drafted the manuscript. ST wrote the Excel macro. ASB and TVG conducted the EASE analysis. TVG and MLG provided the microarray data. AJS supervised the analysis. All authors read and approved the final manuscript.

## Supplementary Material

Additional File 1 **Over-represented gene categories in each of the regression patterns and k-means clusters from EASE functional analysis**. "Ease Analysis.xls" contains 2 worksheets: worksheet "regression" contains the over-represented gene categories in each of the 9 regression patterns obtained from EASE functional analysis; worksheet "k-means" contains the over-represented gene categories in each of the 9 k-means clusters obtained from EASE functional analysis. Click here for file

## Figures and Tables

**Table 1:** Type I Sum of Squares used to construct F test for pattern determination.

Type I Sum of Squares	Interpretations	F tests
*SS*(*linear*)	total variability in the experiment due to the linear effect of time	
*SS*(*quadratic *| *linear*)	total variability in the experiment due to the quadratic effect of time that is not contained in *SS*(*linear*)	
*SS*(*residual*)	*SS*(*total*) - *SS*(*linear*) - *SS*(*quadratic *| *linear*)	

*SS*(*total*) is the total variability in the experiment; *df*1, *df*2, and *df*3 represent the degree of freedoms of *SS*(*linear*), *SS*(*quadratic *| *linear*), and *SS*(*residual*), respectively.

**Table 2:** Determination of gene temporal expression patterns by the proposed regression method.

Regression Patterns		Sign of	Sign of	Predicted Signals
Linear	up (LU)	+	N/A	N/A
	down (LD)	-	N/A	N/A
Quadratic	concave (QC)	N/A	-	N/A
	convex (QV)	N/A	+	N/A
Quadratic-Linear	concave up (QLCU)	N/A	-	
	concave down (QLCD)	N/A	-	
	convex up (QLVU)	N/A	+	
	convex down (QLVD)	N/A	+	

"+" if the estimate of  or  is positive, "-" if the estimate of  or  is negative, "N/A" if not applicable,  is the predicted signal at the first time point, and  is the predicted signal at the last time point.

**Table 3:** Comparisons of regression patterns and Peddada et al.'s profiles obtained from 3834 present genes.

Regression patterns	Peddada et al.'s profiles							
	
	MI (48)	MD (16)	UD2 (155)	UD3 (25)	UD4 (34)	DU2 (62)	DU3 (26)	DU4 (13)
LU (228)	41	0	0	0	13	11	0	1
LD (123)	0	11	8	0	0	0	0	0
QC (69)	0	0	14	7	1	0	0	0
QV (15)	0	0	0	0	0	0	3	1
QLCU (20)	2	0	1	3	3	0	0	0
QLCD (214)	0	3	102	9	1	0	0	0
QLVU (125)	2	0	0	0	0	44	13	2
QLVD (4)	0	0	0	0	0	0	0	1

The numbers in the parenthesis represent the numbers of genes contained in regression patterns or Peddada et al.'s profiles obtained from the analyses on the data of 3834 genes.

**Table 4:** Comparisons of regression patterns and k-means clusters obtained from 770 ANOVA significant genes.

Regression Patterns	K-means Clusters								
	
	K1 (163)	K2 (126)	K3 (107)	K4 (42)	K5 (81)	K6 (95)	K7 (41)	K8 (64)	K9 (51)
FLAT (211)	12	30	36	18	19	8	41	20	27
LU (165)	0	68	0	0	51	10	0	36	0
LD (72)	19	0	53	0	0	0	0	0	0
QC (43)	5	0	0	20	0	0	0	0	18
QV (8)	0	1	0	0	0	7	0	0	0
QLCU (13)	0	0	0	0	4	0	0	8	1
QLCD (151)	127	0	15	4	0	0	0	0	5
QLVU (104)	0	27	0	0	7	70	0	0	0
QLVD (3)	0	0	3	0	0	0	0	0	0

The numbers in the parenthesis represent numbers of genes contained in regression patterns or k-means clusters obtained from the analyses on the data of 770 genes.

**Table 5:** Over-represented gene categories in some regression patterns from EASE functional analysis.

**Reg. Patterns**	**Gene Category**	**List Hits**	**List Total**	**Pop. Hits**	**Pop. Total**	**EASE Score**
**LD**	**Cell adhesion**	10	64	52	699	3.53E-02
**LU**	**Immune regulation**					
	defense response	43	159	98	699	9.77E-07
	response to biotic stimulus	44	159	104	699	2.38E-06
	immune response	37	159	88	699	2.77E-05
	response to external stimulus	50	159	142	699	1.79E-04
	immune cell activation	5	159	6	699	2.58E-02
	cell activation	5	159	6	699	2.58E-02
	lymphocyte activation	5	159	6	699	2.58E-02
	B-cell activation	4	159	4	699	3.79E-02
	**Other biological functions**	18	159	43	699	7.52E-03

**QLCD**	**Coenzyme and prosthetic group metabolism**	7	139	12	699	1.73E-02

**QLCU**	**Signaling**					
	cyclic-nucleotide-mediated signaling	3	12	4	699	1.33E-03
	second-messenger-mediated signaling	3	12	5	699	2.20E-03
	G-protein signaling, coupled to cyclic nucleotide second messenger	2	12	3	699	4.65E-02
	cAMP-mediated signaling	2	12	3	699	4.65E-02
	**Protein metabolism**	7	12	173	699	3.16E-02

**QLVU**	**Immune regulation**					
	response to pest/pathogen/parasite	21	98	61	699	5.80E-05
	response to wounding	14	98	40	699	1.54E-03
	inflammatory response	12	98	32	699	2.19E-03
	innate immune response	12	98	32	699	2.19E-03
	defense response	24	98	98	699	3.88E-03
	response to biotic stimulus	25	98	104	699	3.98E-03
	immune response	22	98	88	699	4.82E-03
	response to stress	23	98	98	699	8.67E-03
	response to chemical substance	7	98	18	699	2.80E-02
	humoral defense mechanism (sensu Vertebrata)	6	98	14	699	3.32E-02
	response to external stimulus	28	98	142	699	3.53E-02
	**Cell surface receptor linked signal transduction**	18	98	80	699	3.64E-02
	**Cell-matrix adhesion**	4	98	6	699	3.78E-02

"Reg. Patterns" stands for the regression patterns identified by the proposed regression method; in the "Gene Category" column, the gene categories are further summarized to broader categories (in bold); "Pop. Total" stands for the number of total input genes (770) that are contained in EASE database, the remaining 71 genes do not have a biological function identified by EASE; "Pop. Hits" stands for the number of genes in "Pop. Total" that are classified into each gene category; "List Total" stands for the number of genes in "Pop. Total" that are classified into each regression pattern; "List Hits" stands for the number of genes in "List Total" that are classified into each gene category.

**Table 6:** Over-represented gene categories in some k-means clusters from EASE functional analysis.

**K-means Clusters**	**Gene Category**	**List Hits**	**List Total**	**Pop. Hits**	**Pop. Total**	**EASE Score**
**K2**	**Immune Regulation**					
	immune response	23	121	88	699	3.02E-02
	defense response	25	121	98	699	3.02E-02
	response to biotic stimulus	26	121	104	699	3.38E-02

**K4**	**Humoral immune regulation**	5	37	24	699	3.01E-02

**K5**	**Immune Regulation**					
	immune response	18	80	88	699	1.25E-02
	response to biotic stimulus	20	80	104	699	1.51E-02
	defense response	19	80	98	699	1.72E-02
	**Cell death**					
	apoptosis	9	80	34	699	2.91E-02
	programmed cell death	9	80	35	699	3.43E-02
	**Ion Homeostasis**					
	ion homeostasis	5	80	13	699	4.88E-02
	cell ion homeostasis	5	80	13	699	4.88E-02
	**Embryogenesis and morphogenesis**	4	80	6	699	2.16E-02
	**Other biological functions**	12	80	43	699	5.39E-03

**K6**	**Immune Regulation**					
	innate immune response	14	87	32	699	3.03E-05
	inflammatory response	14	87	32	699	3.03E-05
	response to pest/pathogen/parasite	20	87	61	699	3.32E-05
	response to wounding	15	87	40	699	1.04E-04
	defense response	24	87	98	699	6.22E-04
	response to biotic stimulus	24	87	104	699	1.57E-03
	immune response	21	87	88	699	2.41E-03
	response to stress	22	87	98	699	4.07E-03
	response to chemical substance	7	87	18	699	1.59E-02
	acute-phase response	4	87	6	699	2.73E-02
	chemotaxis	6	87	16	699	3.65E-02
	taxis	6	87	16	699	3.65E-02
	response to external stimulus	25	87	142	699	4.55E-02
	**Regulation**					
	regulation of biological process	11	87	39	699	1.45E-02
	regulation of cellular process	11	87	39	699	1.45E-02
	regulation of cell proliferation	9	87	30	699	2.25E-02
	**Cell surface receptor linked signal transduction**	17	87	80	699	2.50E-02

In the "Gene Category" column, the gene categories are further summarized to broader categories (in bold); "Pop. Total" stands for the number of total input genes that are contained in EASE database; "Pop. Hits" stands for the number of genes in "Pop. Total" that are classified into each gene category; "List Total" stands for the number of genes in "Pop. Total" that are classified into each k-means cluster; "List Hits" stands for the number of genes in "List Total" that are classified into each gene category.
